# Attitudes about donor information differ greatly between IVF couples using their own gametes and those receiving or donating oocytes or sperm

**DOI:** 10.1007/s10815-016-0694-4

**Published:** 2016-04-08

**Authors:** Agneta Skoog Svanberg, G. Sydsjö, M. Bladh, C. Lampic

**Affiliations:** Department of Women’s and Children’s Health, Uppsala University, SE-751 85 Uppsala, Sweden; Obstetrics and Gynecology, Department of Clinical and Experimental Medicine, Faculty of Health Sciences, Linköping University, Linköping, Sweden; Department of Gynecology and Obstetrics in Linköping, County Council of Östergötland, Linköping, Sweden; Department of Neurobiology, Care Sciences and Society, Karolinska Institutet, SE-141 83 Huddinge, Sweden

**Keywords:** Attitudes, Gamete donation, Embryo donation

## Abstract

**Objective:**

The objective of the study is to examine attitudes towards aspects of donation treatment based on a national Swedish sample of gamete donors and couples undergoing assisted reproductive techniques (ART).

**Methods:**

The present study was part of the Swedish study on gamete donation, a prospective longitudinal cohort study including all fertility clinics performing gamete donation in Sweden. The sample comprised 164 oocyte donors, 89 sperm donors, 251 people treated with their own gametes (in vitro fertilisation (IVF)), 213 oocyte recipients and 487 sperm recipients. A study-specific questionnaire was used.

**Results:**

Attitudes vary widely between couples using their own gametes for IVF and those receiving or donating oocyte or sperm. The groups differed in their responses to most questions. Oocyte and sperm donors were more likely to agree with the statements “The donor should be informed if the donation results in a child” and “Offspring should receive some information about the donor during mature adolescence” than recipients of donated gametes and couples treated with their own gametes.

**Conclusion:**

Donor recipients, IVF couples and donors expressed different attitudes towards openness and information when it came to gamete donation, and those differences seemed to depend on their current reproductive situation.

## Introduction

The use of assisted reproductive techniques (ART) is increasing around the world. ART are medical treatments that are of interest to medical and legal professionals as well as to the media and society in general. The ethical, medical and psychological consequences of using donated gametes, such as sperm and oocytes, as well as when both are used to form an embryo, are a matter for debate among professionals and society in general.

Sweden was the first country in the world to pass a law in 1985 stipulating that all children born from donated gametes have the right to obtain identifying information about the donor when they are approximately 18 years old [1]. Several countries have followed suit and, at the moment, 11 jurisdictions only allow identifiable donors [2]. Donor programmes vary widely with regard to how much information the donor and the recipient couples can receive about each other, ranging from none or basic information about age and educational level to personal contact. Identity release donor programmes, such as the one in Sweden, guarantee the offspring’s right to information about the donor, while the recipient couple and the donor have no legal right to receive information about each other.

Opinions on gamete donation sometimes differ between the public and medical and legal professionals. In a national population-based study of Swedish gynaecologists, opinions about oocyte and sperm donation varied depending on the healthcare professional’s age and gender [3]. The majority of the gynaecologists thought that donors should not be entitled to information about the recipients’ education and private interests, but 40 % was permissive about providing information about the donor to the parents. The vast majority was in favour of parents being honest with the child about his or her genetic origin.

Lampic and co-workers [3] studied the attitudes about disclosure among professionals working in the Nordic countries, such as doctors, midwives, embryologists and lab technicians. They found great discrepancies between attitudes towards different aspects of donation among Nordic in vitro fertilisation (IVF) doctors and the national legislation in these countries. Norwegian and Danish doctors were concerned that disclosing information to offspring would result in negative outcomes for the family. One in four believed that knowledge about the donor could disturb the child’s relationship with their parents.

A recent study investigated attitudes towards embryo donation among men and women from the general population in a country where embryo donation was not allowed. The results showed that 73 % was positive towards embryo donation, 47 % held the view that that the recipient should be anonymous to the donor and 38 % thought that the donor should remain anonymous to the child [4].

A review by Hudson et al. [5] explored public opinions on gamete donation for infertility treatment. The authors concluded that further research was needed involving a wider range of participants, in order to gain a representative public view on issues such as donation. They said that it was important to find out if people had reservations about the moral, ethical, psychological, legal and financial implication of becoming a donor. If this is the case, then it will become hard to recruit donors in the future. Also, couples who had created their family with the help of donor gametes might be faced with restrictive attitudes from the public and this may affect openness and disclosure. Likewise, couples who have had problems conceiving and are childless might have different opinions from professionals working in the ART area.

We aimed to investigate attitudes towards aspects of donation treatment and future treatment options among couples undergoing ART and gamete donors.

### Participants and procedure

The present study is part of the longitudinal Swedish study on gamete donation, a prospective study of donors and recipients of donated sperm and oocytes, as well as a group of men and women treated with IVF using their own gametes. This multi-centre study included all infertility clinics performing gamete donation in Sweden—that is, clinics at the University hospitals in Stockholm, Gothenburg, Uppsala, Umeå, Linköping, Örebro and Malmö.

Recruitment took place between 2005 and 2008. A consecutive cohort of gamete donors and couples starting donation treatment or IVF using their own gametes were approached about taking part when they were accepted for treatment at the clinic. The only exclusion criteria were people who did not speak or read Swedish. IVF couples using their own gametes were recruited from the University clinics in Uppsala, Linköping and Örebro.

We approached a number of eligible parties involved in ART to take part and the following numbers agreed: 307 of the 477 oocyte recipients, 587 of the 766 sperm recipients, 181 of the 251 oocyte donors, 119 of the 173 sperm donors and 151 of the 212 IVF couples using their own gametes.

**Fig. 1:**
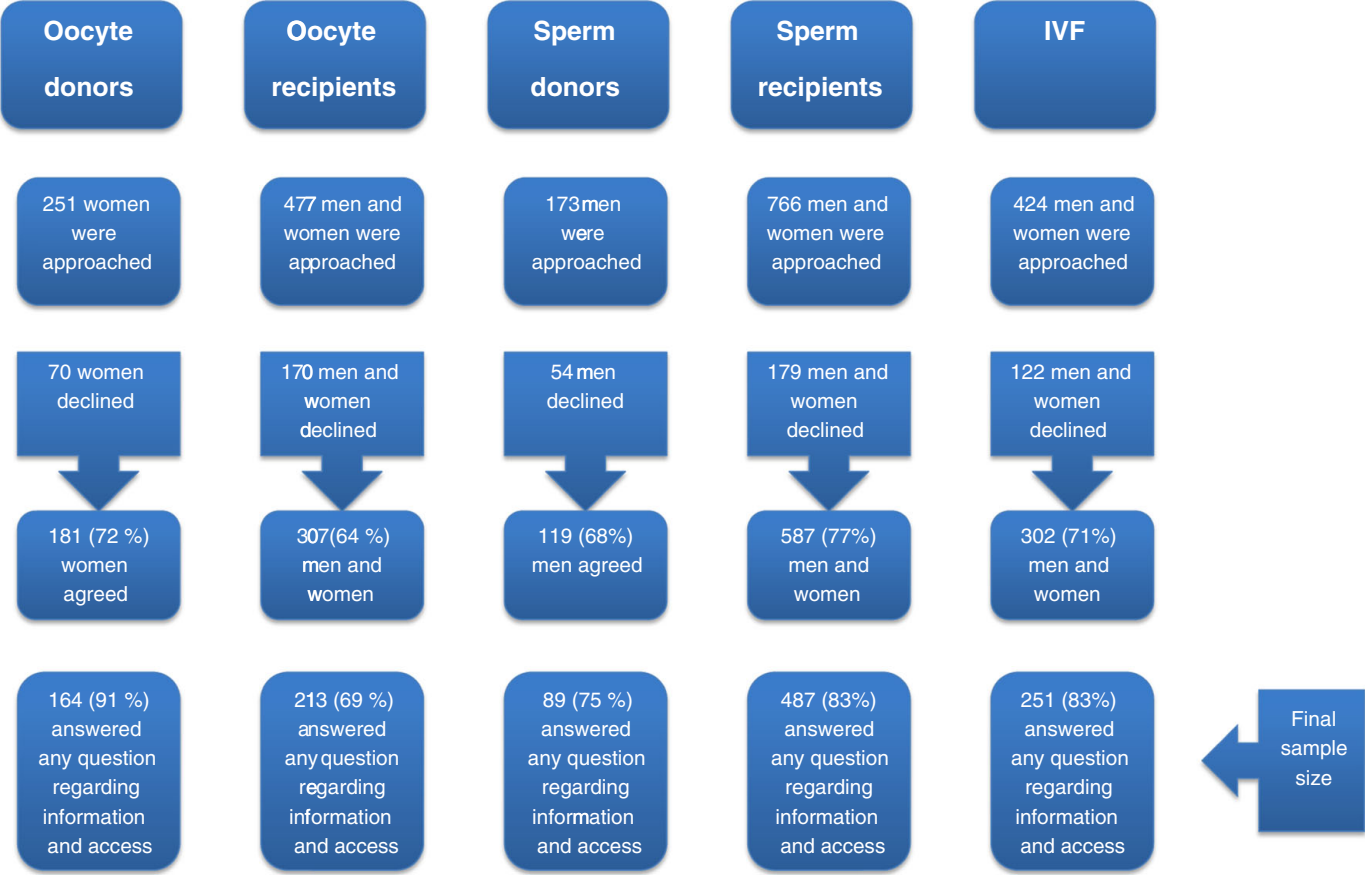
Participants in the study

For this study, participants who had answered any of the questions regarding information and access to gamete donation were included. This resulted in a cohort that included 164 oocyte donors, 89 sperm donors, 251 people treated with their own gametes (IVF), 213 oocyte recipients and 487 sperm recipients (see Fig. 1).

All participants were asked to complete a questionnaire with study-specific questions that assessed their attitudes and opinions on issues concerning access to information and information provided during donation treatment. The questionnaires were distributed together with a prepaid return envelope and a cover letter stating the purpose of the study. Two reminders were sent out to non-respondents and participants were rewarded with gift vouchers worth 12 euros.

### The questionnaire

The attitude questions were developed using statements and response alternatives that had been used in previous studies on attitudes towards different aspects of ART. Different groups have taken part in studies including gynaecologists, medical students, men and women who had recently become parents and IVF healthcare personnel in the Nordic countries [3, 6, 7].

Questions about age, education and number of children were included, as well as six questions measuring their opinions on donations. The response alternatives for the six attitude questions were: completely agree, agree, neutral, agree to some extent, do not agree and indecisive. The study participants were asked if:

- The donor should be informed if the donation resulted in a child.
- The donor should receive some information about the recipients, such as their education and interests.
- The recipients should receive some information about the donor, such as their education and interests.
- Offspring should receive some information about the donor during childhood.
- Offspring should receive some information about the donor during mature adolescence.
- Embryo donation should be allowed.

### Statistics

Pearson’s chi-square was used to investigate bivariate differences between attitudes and background variables. A multi-nomial logistic regression, which is an extension of the binary logistic regression model and allows for more than two levels on the outcome variable, was performed to evaluate if significant bivariate differences vanished when they were also adjusted for all background variables. Outcomes for each of the six attitudes were measured and predictors were age (≤30 or >30 years), education (elementary school or high school, university), gender (women/treated partner or men/accompanying partner) and group (oocyte donors, oocyte recipients, sperm donors, sperm recipients or traditional IVF). The answer categories for each of the six attitudes were recoded into agree, disagree, neutral and indecisive.

All statistical analyses were performed using the IBM SPSS programme, version 20.0 (IBM Corp, Armonk, NY, USA). Due to multiple testing, a *p* value <0.01 was considered statistically significant.

## Results

The participants’ socio-demographic data are displayed in Tables 1 and 2. As expected, the participants differed in most of the variables, except for the comparison of sperm recipients and IVF-treated couples, where no differences were detected.

**Table 1:** Background characteristics of the participants, numbers and percentages

	Oocyte donors		Oocyte recipients		Sperm donors		Sperm recipients		IVF with own gametes	
	*n*	%	*n*	%	*n*	%	*n*	%	*n*	%
Age										
≤30	68	41.5	36	16.9	35	39.3	153	31.4	75	29.9
>30	96	58.5	177	83.1	54	60.7	334	68.6	176	70.1
Education										
Elementary school	7	4.3	18	8.5	0	0.0	18	3.7	17	6.9
High school	81	49.7	101	47.6	28	31.5	209	43.2	104	42.4
University	75	46.0	93	43.9	61	68.5	257	53.1	124	50.6
Gender										
Women/treated partner	164	100.0	110	51.6	0	0.0	244	50.1	125	49.8
Men/accompanying partner	0	0.0	103	48.4	89	100.0	243	49.9	126	50.2
Previous children										
Yes	110	67.1	20	9.4	31	34.8	55	11.3	23	9.2
No	54	32.9	193	90.6	58	65.2	432	88.7	228	90.8

**Table 2:** Background characteristics of the participants, statistical significance

	Oocyte donors vs. oocyte recipients	Oocyte donors vs. IVF with own gametes	Oocyte recipients vs. IVF with own gametes	Sperm donors vs. sperm recipients	Sperm donors vs. IVF with own gametes	Sperm recipients vs. IVF with own gametes
	*p* value	*p* value	*p* value	*p* value	*p* value	*p* value
Age	<0.001	0.015	0.001	0.143	0.102	0.689
Education	0.271	0.257	0.345	0.011	0.003	0.155
Gender	–	–	0.692	–	–	0.938
Previous children	<0.001	<0.001	0.933	<0.001	<0.001	0.373

Tables 3 and 4 show the attitudes towards information on, and access to, gamete donation by the different groups of participants: oocyte donors, oocyte recipients, sperm donors, sperm recipients and couples treated with their own gametes. The different groups of participants differed when it came to most attitudes. Oocyte and sperm donors were more likely to agree with the statements “The donor should be informed if the donation resulted in a child” and “Offspring should receive some information about the donor during mature adolescence” compared to oocyte and sperm recipients and couples treated with their own gametes. Comparing all groups, oocyte donors were the least likely to agree to the statement “Embryo donation should be allowed”. Overall, the fewest numbers of statistically significant differences occurred when comparing sperm donors with sperm recipients.

**Table 3:** Attitudes towards information about, and access to, gamete donation by study group presented as number as percentages

		Group				
		Oocyte donors	Oocyte recipients	Sperm donors	Sperm recipients	IVF with own gametes
		(*n* = 159–164) (%)	(*n* = 208–213) (%)	(*n* = 86–89) (%)	(*n* = 476–486) (%)	(*n* = 247–251) (%)
The donor should be informed if the donation resulted in a child	Agree	89.0	60.1	73.0	38.9	29.9
	Neutral	4.9	8.5	12.4	12.9	10.4
	Disagree	1.8	23.0	11.2	38.3	44.2
	Indecisive	4.3	8.5	3.4	10.3	15.5
The donor should receive some information about the recipients, such as education and interests	Agree	12.2	16.9	13.5	9.5	17.1
	Neutral	18.3	10.3	10.1	7.6	9.6
	Disagree	64.0	67.1	73.0	75.5	60.6
	Indecisive	5.5	5.6	3.4	7.4	12.7
The recipients should receive some information about the donor, such as education, and interests	Agree	32.1	30.0	43.8	35.5	45.2
	Neutral	14.8	10.3	11.2	8.3	12.0
	Disagree	46.9	55.4	38.2	51.9	34.8
	Indecisive	6.2	4.2	6.7	4.3	8.0
Offspring should receive some information about the donor during childhood	Agree	28.3	24.5	23.3	20.3	19.8
	Neutral	15.7	7.7	19.8	10.1	11.3
	Disagree	42.1	59.1	44.2	55.2	44.1
	Indecisive	13.8	8.7	12.8	14.4	24.7
Offspring should receive some information about the donor during mature adolescence	Agree	72.8	50.0	72.4	52.9	35.9
	Neutral	8.6	8.8	9.2	8.6	Tta 10.6
	Disagree	9.3	31.9	6.9	25.7	30.2
	Indecisive	9.3	9.3	11.5	12.8	23.3
Embryo donation should be allowed	Agree	31.7	52.4	46.6	45.7	44.6
	Neutral	5.6	7.1	8.0	5.1	8.8
	Disagree	16.8	18.1	4.5	9.7	15.1
	Indecisive	46.0	22.4	40.9	39.5	31.5

^1^Oocyte donors vs. oocyte recipients

^2^Oocyte donors vs. IVF with own gametes

^3^Oocyte recipients vs. IVF with own gametes

^4^Sperm donors vs. sperm recipients

^5^Sperm donors vs. IVF with own gametes

^6^Sperm recipients vs. IVF with own gametes

**Table 4:** Statistical significance, *p* value, on difference in attitudes towards information about, and access to, gamete donation by study group

	Oocyte donors vs. oocyte recipients	Oocyte donors vs. IVF with own gametes	Oocyte recipients vs. IVF with own gametes	Sperm donors vs. sperm recipients	Sperm donors vs. IVF with own gametes	Sperm recipients vs. IVF with own gametes
The donor should be informed if the donation resulted in a child	<0.001	<0.001	<0.001	<0.001	<0.001	0.022
The donor should receive some information about the recipients, such as education and interests	0.123	0.005	0.072	0.301	0.051	<0.001
The recipients should receive some information about the donor, such as education, and interests	0.307	0.031	<0.001	0.116	0.938	<0.001
Offspring should receive some information about the donor during childhood	0.005	0.018	<0.001	0.049	0.048	0.003
Offspring should receive some information about the donor during mature adolescence	<0.001	<0.001	<0.001	0.001	<0.001	<0.001
Embryo donation should be allowed	<0.001	0.011	0.118	0.346	0.052	0.014

The multi-nomial logistic regression of agree versus indecisive, where adjustments were made for age, education, gender and group, found that oocyte donors and sperm donors were more than 10 times more likely to agree to the statement “The donor should be informed if the donation resulted in a child” than people treated with their own gametes. Also, individuals who had been treated for infertility with donated gametes, or who had donated gametes, were more likely to agree to the statements “Offspring should receive some information about the donor during childhood” and “Offspring should receive some information about the donor during mature adolescence” compared to individuals treated with their own gametes (Table 5). However, no statistically significant differences between groups were found for the statement “The recipient should receive some information about the donor, such as education and interests”.

**Table 5:** Multinomial logistic regression adjusted for gender, education and age

		Agree vs. indecisive	Disagree vs. indecisive	Agree vs. disagree
The donor should be informed if the donation resulted in a child	IVF	Reference level	Reference level	Reference level
	Oocyte donors	10.28 (4.26–24.80)	0.17 (0.04–0.71)	59.26 (18.00–195.11)
	Sperm donors	11.10 (3.19–38.63)	1.14 (0.29–4.44)	9.80 (4.63–20.72)
	Oocyte recipients	3.87 (2.05–7.59)	1.00 (0.52–1.94)	3.79 (2.42–5.93)
	Sperm recipients	1.94 (1.18–3.20)	1.37 (0.84–2.22)	1.42 (0.99–2.04)
The donor should receive some information about the recipients, such as education and interests	IVF	Reference level	Reference level	Reference level
	Oocyte donors	1.43 (0.55–3.77)	2.57 (1.13–5.84)	0.55 (0.29–1.04)
	Sperm donors	2.55 (0.64–10.24)	4.08 (1.17–14.24)	0.62 (0.29–1.30)
	Oocyte recipients	2.50 (1.11–5.61)	2.80 (1.38–5.69)	0.59 (0.54–1.48)
	Sperm recipients	0.92 (0.49–1.73)	2.16 (1.29–3.62)	0.42 (0.27–0.67)
The recipients should receive some information about the donor such as education and interests	IVF	Reference level	Reference level	Reference level
	Oocyte donors	0.86 (0.35–2.08)	1.79 (0.74–4.31)	0.48 (0.30–0.78)
	Sperm donors	1.08 (0.38–3.07)	1.21 (0.42–3.46)	0.90 (0.51–1.58)
	Oocyte recipients	1.33 (0.56–3.11)	3.28 (1.41–7.60)	0.41 (0.27–0.62)
	Sperm recipients	1.43 (0.74–2.76)	2.74 (1.41–5.32)	0.52 (0.37–0.74)
Offspring should receive some information about the donor during childhood	IVF	Reference level	Reference level	Reference level
	Oocyte donors	2.72 (1.37–5.38)	1.94 (1.05–3.62)	1.38 (0.80–2.36)
	Sperm donors	2.57 (1.08–6.14)	1.86 (0.86–4.04)	1.38 (0.70–2.71)
	Oocyte recipients	3.52 (1.81–6.85)	4.08 (2.25–7.37)	0.87 (0.54–1.40)
	Sperm recipients	1.85 (1.13–3.03)	2.20 (1.45–3.34)	0.84 (0.55–1.27)
Offspring should receive some information about the donor during mature adolescence	IVF	Reference level	Reference level	Reference level
	Oocyte donors	5.35 (2.71–10.57)	0.89 (0.38–2.07)	6.08 (3.19–11.59)
	Sperm donors	4.29 (1.96–9.37)	0.49 (0.16–1.48)	8.69 (3.48–21.67)
	Oocyte recipients	3.53 (1.94–6.43)	2.71 (1.45–5.05)	1.32 (0.84–2.06)
	Sperm recipients	2.71 (1.75–4.20)	1.62 (1.02–2.59)	1.69 (1.15–2.48)
Embryo donation should be allowed	IVF	Reference level	Reference level	Reference level
	Oocyte donors	0.48 (0.29–0.78)	0.78 (0.41–1.46)	0.61 (0.32–1.16)
	Sperm donors	0.86 (0.49–1.51)	0.25 (0.08–1.55)	3.43 (1.21–10.52)
	Oocyte recipients	1.64 (1.04–2.57)	1.67 (0.93–2.99)	0.98 (0.58–1.66)
	Sperm recipients	0.84 (0.59–1.19)	0.53 (0.32–0.88)	1.57 (0.96–2.56)

When analysing the outcome disagree versus indecisive, individuals treated with donated gametes, or who had donated gametes, were more likely to disagree with the statements “The donor should receive some information about the recipients”, “Offspring should receive some information about the donor during childhood” and “Offspring should receive some information about the donor during mature adolescence” than individuals who were treated with their own gametes (Table 5).

When analysing the outcome agree versus disagree, oocyte donors, sperm donors and oocyte recipients were more likely to agree to the statements “The donor should be informed if the donation resulted in a child” compared to individuals treated with their own gametes.

Also, oocyte donors, oocyte recipients and sperm recipients were less likely to agree to the statement “The recipient should receive some information about the donor” compared to individuals treated with their own gametes. Oocyte donors, sperm donors and sperm recipients were more likely to agree to “Offspring should receive some information about the donor during mature adolescence” compared to individuals treated with their own gametes (Table 5).

## Discussion

Lawmakers, public health and healthcare professionals have difficulties formulating clear standpoints on matters that are delicate and do not involve the majority of people in a society. ART and their consequences fall into this category. In the present study, the results clearly showed different attitudes depending on whether the matter was of concern to an individual. Men and women who did not need donated gametes showed a more restricted opinion about donations and openness. The oocyte and sperm donors expressed views on treatment opportunities and openness that differed markedly from those held by donor recipients and IVF couples.

Oocyte donors were least likely to agree or were indecisive about the statement “Embryo donation should be allowed”. Attitudes towards embryo donation have been studied in different populations and in different settings. An Australian study by Kovacs et al. [8] reported that 10 % of the IVF couples with stored embryos would consider donating their embryos. Wånggren et al. [9] found a more permissive attitude among Swedish IVF couples with surplus embryos in storage. The IVF couples were the most permissive about openness concerning information about the donor as well as the couples receiving donated gametes and that the couples receiving the donations should have information about the donors. But men and women in the IVF group displayed a more restrictive attitude to the view that the donor and the child should be given information about each other. It could be that the donors and the recipients of donated gametes were all informed about the regulations and the law at the clinics before being accepted for treatment or donation and they had more knowledge on which to form an opinion. Also, there was a great discrepancy in the attitudes towards certain issues within the groups as well as between the groups. An explanation for this might be that the participants’ attitudes are reflecting their own personal situation in life. This might have an effect on the families and donors openness in the future. The multivariate analyses strengthened these findings.

The legal, psychological and ethical dilemmas on embryo donation might be hard to comprehend for professionals and people who have had the children they wanted and for those who have an infertility problem. For most people, the term embryo donation might be confused with egg donation. Therefore, there may be limited knowledge that the persons donating the embryo are not genetically related to the woman having the embryo transfer and her partner.

There have been a number of studies on patients’ attitudes towards surplus cryopreserved embryos for treatment and research [9–11]. Overall, the results in these studies show that the couples’ attitudes towards donating surplus embryos were not conclusive.

In the present study, conducted at a time when none of the participants had been through their planned treatments or donation, the results showed that it was hard to find a standpoint that the majority of the men and women of reproductive age agreed on.

There is a growing acceptance of gamete donation issues and public acceptance is thought to be increasing. Studying and gaining knowledge about general attitudes towards issues such as embryo donation in the general population is important. Legal and medical professionals need to have research results in order to make predictions about behaviours in the future, but also to have a sense of control on issues that need to be discussed and further studied and explored in a scientific manner.

Attitudes and behaviour are not always compatible. The amount of understanding and knowledge on topics not affecting us or people close to us, such as family members, is often handled more hastily, or the knowledge is too vague to form a considered attitude [5]. Most people feel that it is important to be socially accepted and to feel that their attitudes are accepted by their social group [5]. Most of us develop attitudes similar to those held by the societal groups we belong to, and when limited social acceptance has an effect on people’s behaviour and views, this might affect vital issues such as gamete donation and parenthood in families created by donations. Attitudes can help us to organise and structure our experience. Knowing a person’s attitude helps us predict their behaviour. According to the theory of planned behaviour [12], if individuals see recommended behaviour as desirable, and if they think their significant others want them to perform in that way, this results in a higher motivation to behave accordingly. However, these intentions do not always lead to concrete behaviour. Previous investigations have shown that peoples’ behaviour is strongly influenced by their confidence in their ability to perform that behaviour [13]. When we measure attitudes that most people will have an opinion about, such as ART, it should be remembered that an attitude involves a person’s feelings and emotions and these are not always based on knowledge. This is in line with other studies indicating that lawmakers, public health and healthcare professionals have difficulties formulating clear standpoints on matters that are delicate [5].

The response rate in this study was good, included all the university clinics in Sweden and focused on a selection of individuals that had all been accepted for treatment or as gamete donors. As with any survey study, response bias may affect results. In this study, we are missing data from 9 to 31 % of potential responders depending on donor/recipient status. Another limitation of this study was the exclusion of the non-Swedish-speaking participants. The responders were not asked any questions about their knowledge on the issues on gamete donation or embryo donation. Therefore, we have no information about whether all the participants knew that an embryo donation involves gametes from two donors or from a donating couple and that the recipient couples would not have any genetic link to the child.

In summary, recipients and IVF couples and donors expressed different attitudes towards openness and information when it came to gamete donation, and those differences seemed to depend on their current reproductive situation. This finding should be considered when communicating with patients and donors in reproductive settings.
